# The complete chloroplast genome of *Distylium tsiangii* Chun ex Walker, a rare and endangered plant

**DOI:** 10.1080/23802359.2019.1703605

**Published:** 2020-01-08

**Authors:** Lei Xie, Lu Wang, Mingyue Zang, Shuifei Chen, Yao Li, Yanming Fang

**Affiliations:** a Co-Innovation Center for Sustainable Forestry in Southern China, College of Biology and the Environment, Key Laboratory of State Forestry and Grassland Administration on Subtropical Forest Biodiversity Conservation, Nanjing Forestry University, Nanjing, China;; b Research Center for Nature Conservation and Biodiversity, State Environmental Protection Scientific Observation and Research Station for Ecology and Environment of Wuyi Mountains, State Environmental Protection Key Laboratory on Biosafety, Nanjing Institute of Environmental Sciences, Ministry of Ecology and Environment, Nanjing, China

**Keywords:** Chloroplast genome, *Distylium tsiangii*, Hamamelidaceae, phylogenetic analysis

## Abstract

*Distylium tsiangii* Chun ex Walker is an evergreen tree species endemic to China. It has been classified as critically endangered in the Red List of China Higher Plants. Here, we sequenced, assembled, and analyzed the complete chloroplast (cp) genome of *D. tsiangii*. The plastome is 159,125 bp in length, with a typical quadripartite structure and consisting of a pair of inverted repeat (IR) regions (26,220 bp) separated by a large single copy (LSC) region (87,897 bp) and a small single copy (SSC) region (18,788 bp). The overall GC content was 38.00%. A total of 131 genes were annotated, including 37 tRNA genes, 86 protein-coding genes, and eight rRNA genes. Phylogenetic analysis showed that *D. tsiangii* was more closely related to *Parrotia subaequalis*.

*Distylium tsiangii* Chun ex Walker is an evergreen tree species in the family Hamamelidaceae. It is endemic to China and narrowly distributed in Wuling Mountains across Guizhou and Hunan Provinces (Chen et al. 2011). The geographic range of *D. tsiangii* was estimated to be less than 5,000 km^2^, and the natural habitats were severely fragmented. Thus, the species has been classified as critically endangered (CR) in the Red List of China Higher Plants (Qin et al. 2017). However, there was limited research on protection biology of *D. tsiangii*. In this study, we firstly report the complete chloroplast (cp) genome of *D. tsiangii* to provide useful genomic information for further studies on the conservation genetics of the species.

Fresh leaves were collected from a wild individual (diameter at breast height 62.4 cm, tree height 15 m) at Baojing, Hunan Province, China (109°17′28″E, 28°37′49″N). The voucher specimen has been preserved at the Herbarium of Nanjing Forestry University (accession number 19082901). Total DNA extraction and whole genome sequencing on the Illumina Hiseq X Ten platform were conducted by Nanjing Genepioneer Biotechnologies Inc. (Nanjing, China). A total of 21,339,212 clean reads were produced and then used for the *de novo* assembly with NOVOplasty 2.7.2 (Dierckxsens et al. 2016). Gene annotation was performed using the CpGAVAS pipeline (Liu et al. 2012). The obtained sequence was submitted to GenBank under the accession number MN711651.

The complete cp genome of *D. tsiangii* is 159,125 bp in length, with a typical quadripartite structure and consisting of a pair of IRs (26,220 bp) separated by LSC (87,897 bp) and SSC (18,788 bp) regions. The overall GC content of the plastome was 38.00%, while the corresponding values of the LSC, SSC, and IR regions were 36.17%, 32.45%, and 43.08%, respectively. A total of 131 genes were annotated, including 37 tRNA genes, 86 protein-coding genes, and eight rRNA genes. Among those, 113 were unique and 18 (*ndhB, rpl2, rpl23, rps12, rps7, rrn16, rrn23, rrn4.5, rrn5, trnA-UGC, trnI-CAU, trnI-GAU, trnL-CAA, trnN-GUU, trnR-ACG, trnV-GAC, ycf15, and ycf2*) were duplicated in IR regions. Fifteen genes (nine protein-coding genes and six tRNA genes) contained one intron, and three genes (*clpP, rps12*, and *ycf3*) contained two introns.

A Bayesian phylogeny was inferred using MrBayes 3.2.6 (Ronquist et al. 2012) to identify the phylogenetic position of *D. tsiangii* in Hamamelidaceae. Complete cp genome sequences of 10 species from Hamamelidaceae, Cercidiphyllaceae, Altingiaceae and Saxifragaceae were aligned by the HomBlocks pipeline (Bi et al. 2018). The best DNA substitution model (GTR + I + G + F) was determined by ModelFinder (Kalyaanamoorthy et al. 2017). A Markov chain Monte Carlo (MCMC) was run for 1,000,000 generations with two parallel searches using four chains, each starting with a random tree. Trees were sampled every 100 generations. The initial 25% of sampled data were discarded as burn-in. Our results showed that *D. tsiangii* was more closely related to *Parrotia subaequalis* (H.T. Chang) R.M. Hao and H.T. Wei with strong support (posterior probability = 1.0; Figure 1).

**Figure 1.**
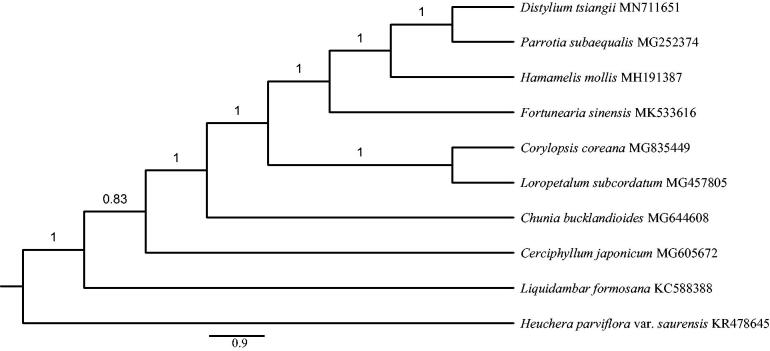
Bayesian phylogeny inferred by MrBayes 3.2.6 (Ronquist et al. 2012) using cp genome sequences of 10 species from Hamamelidaceae, Cercidiphyllaceae, Altingiaceae and Saxifragaceae. The posterior probability value is labeled for each node.
